# An Unexplored Side of Regeneration Niche: Seed Quantity and Quality Are Determined by the Effect of Temperature on Pollen Performance

**DOI:** 10.3389/fpls.2018.01036

**Published:** 2018-07-18

**Authors:** Sergey Rosbakh, Ettore Pacini, Massimo Nepi, Peter Poschlod

**Affiliations:** ^1^Chair of Ecology and Conservation Biology, University of Regensburg, Regensburg, Germany; ^2^Department of Life Sciences, University of Siena, Siena, Italy

**Keywords:** male gametophyte, pollen germination, pollen tube growth, regeneration niche, seed number, seed size

## Abstract

In 1977, Peter Grubb introduced the regeneration niche concept, which assumes that a plant species cannot persist if the environmental conditions are only suitable for adult plant growth and survival, but not for seed production, dispersal, germination, and seedling establishment. During the last decade, this concept has received considerable research attention as it helps to better understand community assembly, population dynamics, and plant responses to environmental changes. Yet, in its present form, it focuses too much on the post-fertilization stages of plant sexual reproduction, neglecting the fact that the environment can operate as a constraint at many points in the chain of processes necessary for successful regeneration. In this review, we draw the attention of the plant ecology research community to the pre-fertilization stages of plant sexual reproduction, an almost ignored but important aspect of the regeneration niche, and their potential consequences for successful seed production. Particularly, we focus on how temperature affects pollen performance and determines plant reproduction success by playing an important role in the temporal and spatial variations in seed quality and quantity. We also review the pollen adaptations to temperature stresses at different levels of plant organization and discuss the plasticity of the performance of pollen under changing temperature conditions. The reviewed literature demonstrates that pre-fertilization stages of seed production, particularly the extreme sensitivity of male gametophyte performance to temperature, are the key determinants of a species’ regeneration niche. Thus, we suggest that previous views stating that the regeneration niche begins with the production of seeds should be modified to include the preceding stages. Lastly, we identify several gaps in pollen-related studies revealing a framework of opportunities for future research, particularly how these findings could be used in the field of plant biology and ecology.

## Introduction

In 1977, Peter Grubb introduced the concept of the regeneration niche, defined as the requirements sexually mature plants have for successful reproduction, dispersal, germination and seedling establishment (Grubb, 1977; Bykova et al., 2012). The beauty of this concept lies in its simplicity – the regeneration niche directly determines species niche, because even if the environmental conditions are suitable for plant growth and survival, a plant species cannot persist if the conditions are not suitable for successful re-establishment and dispersal. Despite several gaps in the field (e.g., a very strong focus on seed morphological traits; Jiménez-Alfaro et al., 2016), the role of the regeneration niche in determining plant occurrence and abundancy at various scales is now commonly recognized in plant ecological research. Yet, the supporters of this concept tend to place too much emphasis on the post-fertilization stages of plant sexual reproduction and assume that the production of sufficient numbers of viable seeds (an essential prerequisite for successful dispersal, seed germination, and post-germination establishment) remains unaffected by abiotic or biotic factors. However, the environment can operate as a constraint during many of the stages necessary for successful fertilization (Hedhly et al., 2009; Zinn et al., 2010). For example, if ambient environmental conditions are unsuitable, one or more steps between gametogenesis to the fusion of male and female gametes may be blocked leading to an irregular, reduced, or missing seed set. During the short time necessary for fertilization, even a single day with extreme weather conditions can be fatal to the reproductive success of many plant species (Zinn et al., 2010). We, therefore, argue that other stages of the plant reproductive cycle (gametogenesis, pollination, fertilization, and seed ripening) are as important as the post-fertilization stages and should be included in the “regeneration niche" concept.

The aim of this review is to draw the attention of the plant ecology research community to the pre-fertilization stages of plant sexual reproduction and their potential importance for successful seed production. More specifically, we focus on how temperature, one of the most concerning environmental factors today, determines qualitative and quantitative differences in seed production due to its effect on the performance of male gametophytes (Barnabas et al., 2008; Zinn et al., 2010; Hedhly, 2011). First, we provide a short summary on the role of seeds in the life cycle of plants, review temporal and spatial variations in seed quality and quantity, followed by a discussion on the role temperature plays on pollen performance within these variations. Then, we briefly review known pollen adaptations to temperature stresses at different levels of plant organization and discuss the plasticity of pollen performance under changing temperature conditions. Finally, we briefly review numerous historical and recent studies on pollen biology and ecology, many of which are currently unknown in the field of functional plant trait ecology. Based on this review, we identify several gaps in pollen-related studies revealing a framework for opportunities in future research, particularly how these findings could be used in the field of plant biology and ecology. We did not review all the available literature, because it would be a monumental task; rather, we focus on representative studies for each of the topics discussed in the current review. Where available, examples of wild plants (i.e., non-cultivated plants growing under natural conditions) are given.

## The Crucial Role of Seeds in Plant Life Cycle

Seeds play several pivotal roles in plant biology and ecology. They provide plant adaptability to the environment by maintaining high genetic flexibility, due to gene recombination during meiosis and the random combination of gametes during fertilization (Fenner, 2000). Seeds can also contribute considerably to the replacement of adults in a plant community that are removed due to senescence, herbivory, or pathogens (Grubb, 1977). Moreover, their comparatively low weight and small size allow seeds to disperse and colonize new areas or gaps in existing communities (Bonn and Poschlod, 1998; Poschlod et al., 2013). Seeds also have higher resistance to environmental stresses (desiccation, low- and high-temperature stress, herbivory in some cases, etc.), when compared to the parent plants, since they can persist in the soil for a long time due to dormancy and a period of arrested development (Thompson et al., 1993, 1997), up to several hundred years in some cases (Sallon et al., 2008). Soil seed banks are therefore an important component of plants’ persistence strategy to a varying environment, especially in short-lived species (Saatkamp et al., 2014). Finally, seeds and fruits are a vital food source for humans and nature, and many species are dependent on their sexual reproduction.

The number and quality of seeds produced by a plant are important determinants of its life history, strategy, population dynamics, persistence, spatial distribution, and many other processes (Grubb, 1977; Grime, 2002; Jump and Woodward, 2003). For instance, if a plant species is able to produce more sound seeds (e.g., higher seed filling rates or larger seeds), then it can, for instance, be more abundant in plant communities (Turnbull et al., 2000) and have greater chances to colonize and establish itself in new areas (Henrik Bruun and Poschlod, 2006). Higher quality seeds can also increase recruitment (Moles and Westoby, 2002), increase competitive advantage (Bazzaz et al., 1989), increase chances of survival under stressful conditions (Walters and Reich, 2000), and persist longer in the soil (O’Connor and Pickett, 1992). Conversely, low seed production and/or low qualities of seeds negatively affect these processes (e.g., Pigott, 1992; Jacquemyn et al., 2001). However, there is a great deal of variation among plants in seed quantity and quality caused by both evolutionary and ecological factors. As for the former, seed size among species varies from less than 1 μg in some tropical orchids to the enormous 25-kg seeds of coco de mer (double coconut, *Lodoicea maldivica*) (Blackmore et al., 2012). The number of seeds also represents a broad spectrum from the above-mentioned coco de mer concentrating all its reproductive output in 4–11 seeds to the several thousand seeds per capsule in species from the genera *Drosera*, *Orobanche*, *Papaver*, and some orchids (Salisbury, 1942). As for the ecological factors, it has been accepted through recorded history that seed quality is strongly correlated to environmental conditions. Evidence from the vast body of previous research suggests that spatial and temporal environmental variations considerably influence the success of sexual reproduction. To begin with, temporal (e.g., interannual) variations in temperature (Hofgaard, 1993; Kullman, 1993) and precipitation (Erskine and Elashkar, 1993) may result in irregularities in size and quality of seed production in cultivated, as well as in wild species, a phenomenon known as “mast years” (Grubb, 1977). Likewise, spatial environmental variation in temperature, water and nutrient supply, and light intensity at local, regional, and global scales affects both seed quality and seed size, which tend to decrease as environmental favorability declines (Salisbury, 1942; García et al., 2000; Susko and Lovett-Doust, 2000; Jump and Woodward, 2003; Vaupel and Matthies, 2012). Therefore, along with the magnitude of biotic factors affecting plant performance factors, such as intra- and interspecific competition (Westermann and Crothers, 1977; Bell et al., 2005), herbivory (Marquis, 1984), pathogens (Bhaskara Reddy et al., 1999), and pollinators (Bierzychudek, 1981; Burd, 1994), nature itself can have a considerable influence (positive or negative) on the qualitative and quantitative characteristics of the seeds produced.

Although the link between the environment and the success of seed production has been known for a long time, it has received increased interest in last two decades due to the potential consequences of climate change on plants. Specifically, rising average temperatures (Pachauri et al., 2015), along with the increased frequency of extreme weather events, such as hot or cold spells, late frosts and snowfalls, and early snowmelt (Meehl et al., 2000), are of high concern due to their negative effects on seed production in both wild and cultivated plants (Wheeler et al., 2000; Rosenzweig et al., 2001; Rosbakh et al., 2017). Because of their economic importance, these effects have been extensively studied in cultivated plant species (see, e.g., Hedhly et al., 2009; Zinn et al., 2010; Hedhly, 2011 for extensive reviews on the subject). Generally, they include short-term failure of cultivated species, as a direct result of climate instability and a long-term reduction in yields of traditional crops caused by rising average temperatures (Olesen and Bindi, 2002; Peng et al., 2004; Porter and Semenov, 2005; Tubiello et al., 2007). In addition, altered climatic conditions can increase crop vulnerability to infection, pest infestations, and choking weeds, because temperature and water are among the major factors controlling their growth and development (Rosenzweig et al., 2001). Nevertheless, for some cold and temperate regions, the moderate increase in temperature forecasted for the first half of the 21st century is expected to positively affect sexual reproduction of crop species resulting in an increase in average yields (Hedhly et al., 2009). These temperature changes are believed to have a similarly negative influence on seed production in wild populations. Examples of how global warming impacts on the reproductive success of wild plants are much rarer, yet studies show that these impacts will likely promote reproductive success at high latitudes and altitudes and are negative at low latitudes and in lowlands (De Frenne et al., 2010; Klady et al., 2011; Walck et al., 2011; Wagner et al., 2012; Rosbakh et al., 2017). The climate change-induced variation in quality and quantity of seeds produced can potentially lead to altered plant-animal trophic interactions (McKone et al., 1998; Klady et al., 2011), plant population dynamics (Rosbakh et al., 2017), dispersal processes (Walck et al., 2011), persistence in soil (Akinola et al., 1998), and distributional patterns of wild populations (Rosbakh and Poschlod, 2016).

## Temperature Affects the Seed Number Over Male Gametophyte Performance

All aspects of the reproductive cycle are potentially sensitive to climate change (Bykova et al., 2012). For example, the effect of temperature on seed quality and seed number stems from specific physiological limitation in a number of seed developmental stages (Pigott, 1992; Hedhly et al., 2009; Zinn et al., 2010). Although various stages of seed production are temperature-dependent (**Table 1**), numerous experimental studies have demonstrated that pollen, the male gametophyte, is the most temperature sensitive part of the plant sexual reproduction cycle (Mulcahy et al., 1994; Zinn et al., 2010; Steinacher and Wagner, 2012; De Storme and Geelen, 2014).

**Table 1:** Reported temperature (negative) effects on reproduction output in plants.

Stage	Location	Developmental process	Reported effect on seed crops	
			Low-temperature stress	High-temperature stress
Pre-pollination	Androecium	Microsporogenesis	Reduction in seed set seed (Brooking, 1979)	Seed sterility (Sakata et al., 2000)
		Microgametogenesis	Large grain yield reductions (Imin et al., 2006)	Seed sterility (Sakata et al., 2000)
		Anther development	Substantial losses in grain yield (Parish et al., 2012)	Decreased the percentage of fruit set per plant (Sato et al., 2002)
	Gynoecium	Megasporogenesis	NA	Decreased seed and fruit quality (Peet et al., 1997)
		Megagametogenesis	NA	Disrupted seed production (Young et al., 2004)
		Pistil development	Low fertilization success (Huang et al., 2010)	Plants failed to set seed (Polowick and Sawhney, 1988)
Pollination	Stigma	Pollen transfer to stigma	Lower seed set (Totland, 1994)	Reduced seed crop (Mackill et al., 1982)
		Pollen germination	Reduced fruit set (Charles and Harris, 1972)	Spikelet sterility (Rang et al., 2011)
		Stigma receptivity	Reduced pot set (Nayyar et al., 2005)	Reduced fruit set (Charles and Harris, 1972)
Progamic phase	Style	Pollen tube growth	Decrease in seed set (Jakobsen and Martens, 1994)	Lower pod and seed set (Gross and Kigel, 1994)
		Style receptivity		
	Ovary	Pollen tube growth	Decrease in seed set (Jakobsen and Martens, 1994)	Lower pod and seed set (Gross and Kigel, 1994)
		Ovule receptivity	Altered fruit set (Sanzol and Herrero, 2001)	Poor fruit set (Cuevas et al., 1994)
Fertilization	Ovary	Gamete fusion	NA	Lower pod and seed set (Gross and Kigel, 1994)
Post-fertilization	Carpel	Seed maturation	Severe reduction in seed yield (Kaur et al., 2008)	Decline in seed germination, vigor, and physical quality (Keigley and Mullen, 1986)
NA, studies on this aspect are not available.				

Pollen is extremely susceptible to temperature stress as it makes its way to its female counterpart (**Table 2**). Its vulnerability is exacerbated by its comparatively small size, haploid set of chromosomes, lack of protective tissue, and direct exposure to the environment (Bedinger, 1992; Pacini and Dolferus, 2016). In general, a stressful environment restricts plant growth and flower production resulting in reduction of the number and quality of pollen produced (Schlichting, 1986; De Storme and Geelen, 2014).

**Table 2:** Reported temperature negative effects on pollen performance.

Stage	Developmental process	Low-temperature stress	High-temperature stress
Pre-pollination	Microsporogenesis	Parish and Li, 2010	Endo et al., 2009; Parish and Li, 2010
	Microgametogenesis	Jacobs and Pearson, 1999	Pereira et al., 2014
	Anther development	Mamun et al., 2006	Saini et al., 1984
Pollination	Pollen transfer to stigma	NA	NA
	Pollen adhesion	NA	Hedhly et al., 2003
	Pollen–pistil interaction	NA	Hopper et al., 1967; Ascher and Peloquin, 1970; Wilkins and Thorogood, 1992.
	Pollen germination	NA	Kakani et al., 2002; Rang et al., 2011; Sunmonu and Kudo, 2015
	Pollen tube growth	Pasonen et al., 2000	Pasonen et al., 2000; Kakani et al., 2002
Progamic phase	Fertilization	Gerassimova-Navashina et al., 1968	NA
NA, studies on this aspect are not available.			

Pollen performance refers to the traits of the male gametophyte that influence its ability to achieve its highly specialized functions such as: reaching a stigma, germinating, developing a pollen tube that is able to reach the base of the style and enter the ovary, and locating and entering the ovule to deliver sperm to a receptive egg and central cell (Williams and Mazer, 2016). At the very early development stages in the anther, extreme temperatures can negatively affect both proper gametophyte development and the sporophytic anther wall layers leading to pollen sterility, reduced pollen production, or reduced anther dehiscence (Sato et al., 2002; Endo et al., 2009; Parish and Li, 2010). Thus, low- or high-temperature damage occurring during anthesis is likely to induce failure of pollination and/or fertilization, resulting in a lower number of seeds (Lankinen, 2001; Kakani et al., 2005; Lora et al., 2012). Once pollen grains are formed, they are released into the environment after anther dehiscence. This process can be affected by temperature stress, preventing the pollen from leaving the anther and triggering male sterility (Matsui and Omasa, 2002; De Storme and Geelen, 2014). After being released from the anthers, pollen grains need to be dispersed to the immobile female gamete through the hostile environment. In some cases, pollen must wait for a dispersal vector in the open anther for up to several weeks in some orchid species with highly specialized pollination systems (Dafni and Firmage, 2000). Mature pollen grains are thought to be more tolerant to temperature stress than any other stage of male gametophyte development, due to their low cytoplasm water content, low metabolic activity, and carbohydrate contents and dynamics (Hedhly, 2011). However, inclement weather conditions during pollen dispersal are often associated with reduced pollen viability or male sterility (Aronne, 1999; Kakani et al., 2005; Koubouris et al., 2009). At the next stage, pollen adhesion, temperature can also affect further pollen development (Hedhly et al., 2003). Before the re-hydrated pollen grain proceeds to the next step of pollen germination (PG), it must pass the pollen-female counterpart recognition barrier, an important mechanism promoting outcrossing and genetic variability within and among plant populations (Seavey and Bawa, 1986; Castric and Vekemans, 2004). Reportedly, temperature stress affects pollen–pistil interactions during this stage, resulting in the alleviation of self-incompatibility, at least under experimental conditions (Hopper et al., 1967; Ascher and Peloquin, 1970; Wilkins and Thorogood, 1992). Once the pollen has overcome the self-incompatibility barrier, it germinates (binding to the stigma) and develops a pollen tube that continues its growth into the style; the former process has also been shown to be temperature sensitive (Mohammed and Tarpley, 2009; Rang et al., 2011). Furthermore, pollen tubes are also highly susceptible to temperature fluctuations despite growing into the style, where the male gametophyte is presumably better protected from a severe environment by several layers of pistil tissue (Kakani et al., 2002; Ledesma and Sugiyama, 2005). Lastly, the fusion of male and female gametophytes is also sensitive to temperature. For example, prolonged exposure of pollinated *Crepis capillaris* (L.) wallflowers to low temperatures (1, 5, and 8°C) led to the inability of sperm cells to penetrate the female cells, or the failure of nuclei fusion (Gerassimova-Navashina et al., 1968). Consequently, all these potential deviations from the normal course of male gametophyte development can alter reproductive ability by reducing the number of pollen grains deposited on the stigma (Baskin and Baskin, 2015) as well as their viability, which inevitably leads to irregular and reduced seed yields (Zinn et al., 2010; Hedhly, 2011).

## Pollen Paternal Effect on Seed Quality

Apart from the negative effects on seed number caused by temperature stress at different stages of pollen development, altered pollen performance can also influence seed quality which can also affect the characteristics of offspring, such as growth rates, stress tolerance, and competitive ability (Bazzaz et al., 1989; Pigott, 1992; Walters and Reich, 2000; Jacquemyn et al., 2001; Moles and Westoby, 2002). However, the influence of the male gametophytes on seed fitness is a recent paradigm shift in evolutionary ecology on determinants or reproductive success in plants. Until the late 1980s, it was believed that only the maternal environments determined the phenotype of their offspring’s vigor and that characteristics, such as successful capture of pollen and maturation of seeds, came solely from the female gametes (Schlichting, 1986; Mazer and Gorchov, 1996). This view was held because two-thirds of the endosperm and cytoplasmic genetic material originates from the maternal plant and the female plant provisions the seed and determines the thickness of the seed coat. It is these genetic and phenotypical effects that were thought to determine seed size, dormancy, dispersal, longevity, and dispersal rates. Additionally, these maternal effects were also thought to influence germination and establishment rates, as well as life traits through subsequent generations (Stephenson, 1981; Roach and Wulff, 1987; Case et al., 1996; Meyer and Allen, 1999; Galloway, 2001; Kochanek et al., 2010). However, some (though very limited) evidence suggests that even though pollen invests a comparatively small amount of energy in seed production (Stephenson and Bertin, 1983), it may play at least an equal role in determining seed vigor and performance (Schlichting, 1986; Galloway, 2001). The paternal environment, including light, soil nutrients, competition, herbivory, and more importantly temperature, was found to determine their life history, in at least some species, by influencing seed characteristics, such as seed mass, germination time, and the percentage of germination (Schlichting, 1986; Young and Stanton, 1990; Delph et al., 1997; Galloway, 2001; Etterson and Galloway, 2002). Even more intriguing, paternal effects were seen in the later developmental stages as seedlings and adults (e.g., McKenna and Mulcahy, 1983). However, the influence of the paternal environment appears to vary across maternal environments, suggesting that paternal environmental effects still should be evaluated in the context of maternal environments (Schlichting, 1986; Mazer and Gorchov, 1996; Galloway, 2001; Etterson and Galloway, 2002).

There are several paternal environmental and genetic effects on progeny performance that could be caused by abiotic stress, including temperature (reviewed in detail by Mazer and Gorchov, 1996; Delph et al., 1997; Hedhly, 2011). For example, the environmental effects may be caused by the availability of resources (e.g., water, nutrient, light, or any other limiting resource) to the paternal plant which could influence pollen resource provisioning, a direct determinant of pollen quality and competitive ability, resulting in different levels of reproductive success (Schlichting, 1986; Young and Stanton, 1990; Stephenson et al., 1992; Lau and Stephenson, 1994). Progeny from the same paternal plants but different environment may also create different phenotypes and different level of successes due to an environment-specific gene expression induced in the male gametophyte (Galloway, 2001). Moreover, different external environments, such as temperature (Johannsson and Stephenson, 1998), may impose distinct selective regimes on male gametophyte. Thus, if selection among developing male gametophytes is sufficiently strong and environment specific, the genetic composition of mature pollen in a population will depend upon the paternal environment resulting in phenotypically and genetically distinct paternal sibships (Searcy and Mulcahy, 1986; Mazer and Gorchov, 1996). Moreover, paternal environmental effects could also be due to post-pollination selection among male gametophytes in pollen tubes. This highly controversial (Hedhly et al., 2009; Baskin and Baskin, 2015) hypothesis was initially proposed by Mulcahy (1979), and is based on the assumption that high pollen loads deposited on the stigma create a competitive arena for male gametophytic selection (MGS). According to this concept, pollen tubes of pollen grains better adapted to the ambient environmental conditions grow faster and thus fertilize more ovules than the slower growing pollen tubes of the less fit counterparts. Conversely, damaged or maladapted haploid individuals are eliminated, and the genes of the fittest pollen grains are passed on to the next sporophytic generation. If these genes are selected during this process and expressed in the sporophyte (Tanksley et al., 1981; Borges et al., 2008), they could also influence the phenotype of the diploid progeny (McKenna and Mulcahy, 1983; Stephenson and Bertin, 1983; Mazer and Gorchov, 1996; Baskin and Baskin, 2015). Evidence for differential selection at the gametophytic level in response to temperature stress was demonstrated in a few plant species. In an example, offspring derived from gametes that underwent intense pollen competition may demonstrate a greater ability to cope with low- and high-temperature stress (for detailed reviews on this topic, see Hedhly, 2011; Baskin and Baskin, 2015). The ability of gametophyte selection to modify the sporophyte portion of the plant life cycle has important implications for plant population dynamics in a changing environment. However, despite a brief increase in interest on the topic in 1970–1980s, there have only been a few studies published on MGS in last 30 years, and still remains poorly investigated (Baskin and Baskin, 2015). The MGS might serve as an important mechanism of rapid plant adaptation to a changing environment by using natural selection of the best-adapted pollen tubes during the reproductive phase to the prevailing conditions, thereby changing gene frequencies between generations (Hedhly et al., 2009). In wild populations, it could facilitate plant adaptation to rising average temperatures, and the increased frequency of extreme temperatures, by the production of better-adapted progeny. Furthermore, because the high number of pollen grains placed upon a stigma is a crucial prerequisite for MGS to take place, it could help us better understand how pollen limitation, population size, and habitat fragmentation affect the natural processes of plant adaptation to fluctuating environmental conditions. As for cultivated plants, MGS can be an effective tool for (rapid) breeding of landraces (Hedhly et al., 2009) that are well adapted to new temperature conditions, since the selection pressure can be easily applied to the pollen during PG and pollen tube growth (PTG), thus making it a complementary strategy to traditional sporophyte-based breeding strategies (reviewed in Hormaza and Herrero (1996)). Therefore, this particular mechanism should be to the subject of further research.

## Acclimation and Adaptation of Pollen to Temperature Stress at Individual, Population, Species, and Community Levels

Despite the apparently strong effects of temperature variation on male gametophyte performance as mentioned above, plants have developed strategies to cope with such conditions presenting plasticity in their reproductive response to ensure fertilization (Hedhly et al., 2005). These are associated with molecular, physiological, and structural adaptations to particular environmental restraints, as well as the genetic variability of the male gametophyte at different levels.

### Individual Plant Level

Ripe pollen grains that develop normally can respond relatively quickly to short-term low-and high-temperature stress by acclimating their physiological and biochemical processes. In the case of low-temperature stress, the mechanism is well known: unsaturated double-bonds in lipids cause kinks in their hydrophobic tails, reducing their ability to interlock in a frozen lattice (Mulcahy et al., 1994). Furthermore, lowered pollen moisture contents were also demonstrated to protect against imbibitional chilling injuries (Hoekstra, 1984). Furthermore, hydrolysis of complex sugars, particularly inter-conversion of polysaccharides to sucrose and monosaccharides, also helps pollen grains to adjust to freezing temperatures (Vesprini et al., 2002). As for high-temperature stress, pollen is able to synthesize heat shock proteins, in order to protect normal developmental processes (Giorno et al., 2010; Chaturvedi et al., 2016). Reactive oxygen species scavengers, enzymes of carbohydrate metabolism, Ca^2+^ and Ca^2+^-dependent signaling, and specific sugars (e.g., galactinol) are also thought to be involved in pollen heat-tolerance (Frank et al., 2009 and the references therein). However, it is noteworthy that regardless of the type of temperature stress, this acclimation has a comparatively low response rate to the stressor and requires some time before it is initiated (Xiao and Mascarenhas, 1985). For example, soybean pollen tubes will continue to grow at +41°C if the incubation temperature is raised from 29 to 41°C incrementally by 4°C per 15 min. In contrast, pollen germinated at 29°C and suddenly transferred to 41°C is immediately inhibited (Mulcahy et al., 1994). As for long-term fluctuations, interannual variation in temperature conditions (e.g., mean temperature, growth degree days, and number of frost days), especially in cold and temperate climates, strongly affects the flowering phenology of individual plants (e.g., winter-deciduous tree species), and determining the conditions under which fertilization will take place. Therefore, individuals should be able to respond to particular temperature conditions by producing male gametophytes that are adapted to this environment. However, despite being self-evident, to the best of our knowledge, this ability has been demonstrated only once. In an experiment with walnut trees *(Juglans regia* L. cv. Serr), Polito et al. (1991) heated up the individual branches of a single tree over different periods of time (starting from 1 week before the predicted onset of flowering) and evaluated the temperature requirements of PG and PTG. A highly significant correlation (*r*^2^ = 0.996) was found between the optimum temperature for PG and the mean number of degree-days from the start of the experiments to anther dehiscence experienced by the pollen during maturation. Although the authors do not explain the source of this plasticity, genetic (the mechanism of MGS selection; see above), and/or phenotypic (acclimation of pre-formed pollen grains), this finding demonstrates that pollen produced by individual plants can effectively acclimate to current temperature conditions.

Apart from pollen grain adaptations, individual plants demonstrate other anatomical, morphological, and phenological adaptations (mainly in the flower) that help them to adjust the performance of temperature-sensitive male gametophytes to the growing conditions of a micro- and/or macrohabitat. One of the most well-known and most cited adaptations to long-term climatic variations is flower initiation or flowering phenology. Plants tend to flower when the temperature-stress probability is comparatively low (e.g., the majority of plants from Mediterranean climate flower during early spring to avoid the negative effects of high temperatures on reproduction; Bosch et al., 1997). Some varieties of plant growth forms (e.g., cushion and low-stature plants of arctic and upland vegetation) could also be considered as an adaptation facilitating sexual reproduction, since flowers of such plants are located very close to the ground, where local thermic conditions are much more favorable for fertilization processes when compared to the corresponding macrohabitat (Körner, 1999; Geiger et al., 2009). More immediate responses to short-term (e.g., daily) temperature fluctuations include passively or actively modifying intrafloral temperatures (by timing of flower opening-closing, pubescence, heliotropism, and metabolic thermogenesis), anther extrusion, anther dehiscence, and anther opening (Miller, 1986; Stanton and Galen, 1989; Patiño and Grace, 2002; Dietrich and Körner, 2014); for detailed review, see Corbet (1990). Alternatively, the flowering period of a species can last for several weeks or even months, in order to reduce the risk of a mismatch between the temperature requirements of fertilization and the current weather conditions (Lora et al., 2012).

### Variation Within a Plant Population

In natural plant populations, abiotic factors (e.g., illumination, water availability, and soil fertility) are known to vary from one microsite to another. For example, in some spatially heterogeneous habitats, such as alpine grasslands, substantial variation between microhabitats in seasonal mean soil temperature, surface temperature, and season length are typically observed (Scherrer and Körner, 2011). This within-population variation in temperature leads to differences in pollen performance among individuals under different temperature conditions (Pasonen et al., 2000; Lankinen, 2001) and provides an opportunity for the population to ensure sexual reproduction under short-term temperature fluctuations, given that thermal heterogeneity within the population is large enough to affect plant performance (there will always be some individuals siring seeds; Pasonen et al., 2000; Lankinen, 2001). It could also allow adaptation to long-term temperature changes (e.g., global warming) by the selection of pre-adapted male gametophytes from a highly variable pollen pool within the population (Ottaviano et al., 1988; Stephenson et al., 1992).

### Variation Among Several Populations

Since plant species typically consists of several populations located in different environmental conditions, it is expected that the pollen of each population should be adapted to its own local thermal conditions, such as temperature extremes. Indeed, intraspecific variability in male gametophyte response to temperature, exclusively studied in cultivated plants, is well documented and has been used intensively as a selection tool to breed temperature-resistant cultivars (Polito and Weinbaum, 1992; Kakani et al., 2002; Voyiatzis and Paraskevopoulou-Paroussi, 2002; Prasad P.V.V. et al., 2006). For example, several authors found a relationship between the male sporophyte bloom date and low-temperature tolerance of the male gametophyte progeny, with early-blooming individuals producing pollen that is better adapted to germination at lower temperatures (Weinbaum et al., 1984; Luza et al., 1987; Polito et al., 1988).

### Species Level

Spatial and temporal variations in thermal conditions during reproduction vary among different habitats even more widely than the population level, forcing plant species to adapt their pollen performance to local environmental conditions. Species-specific responses of male gametophytes to particular environments have been widely reported for pollen development, anther dehiscence, PG, PTG, and the fusion of the male gametophyte with its female counterpart, including a relatively large number of studies on non-cultivated species (e.g., McKee and Richards, 1998; Harder et al., 2016; Wagner et al., 2017). For example, temperature limits for the normal functioning of progamic processes vary widely from around 0°C for alpine and nival plants (Wagner et al., 2012), to 70°C in *Eucalyptus rhodantha*, an inhabitant extremely hot climates (Heslop-Harrison and Heslop-Harrison, 1985). Additionally, in a multispecies study, Rosbakh and Poschlod (2016) demonstrated that variation in the thermal requirements for PG and PTG among taxa is also strongly associated with their habitat. As for the year-round temperature fluctuations, which can be very drastic in some habitats, Wagner et al. (2017) reported that for most summer-flowering species, PG stopped between 1 and 5°C, whereas the pollen of winter and early spring flowering species germinated at temperatures below zero. Furthermore, germinating pollen was exceptionally frost tolerant in cold adapted plants, but suffered irreversible damage from mild sub-zero temperatures in summer-flowering species (Wagner et al., 2017). Similar findings can be also found in Vesprini and Pacini (2005), Aronne et al. (2006), and Rosbakh and Poschlod (2016).

### Community Level

Ecological theory suggests that local community assembly is shaped through the environment progressively selecting species best adapted to local conditions (de Bello et al., 2013). Some evidence (albeit limited) indicates that the temperature limits of pollen performance may shape species assemblages, at least at larger scales, by selecting for species adapted to the local temperature conditions. Hedhly (2011), in his review on the sensitivity of flowering plant gametophytes to temperature fluctuations, demonstrated that the pollen of species occurring in the same biome share similar optimal temperatures for PG and PTG, ranging from 15 to 25°C for species from temperate regions and optimum temperatures above 25°C for subtropical and tropical species. As for regional scales, the pollen performance of several species typical of high-mountain plant communities in the Alps was found to be similarly robust to low-temperature stress (e.g., cold snaps; Steinacher and Wagner, 2012, 2013). It is therefore suggested that members of the same community, adapted for the same kinds of environments and with the same type of floral biology, will be found to possess comparable pollen traits, once they are investigated (Heslop-Harrison and Heslop-Harrison, 1985).

This evidence on pollen adaptation to particular environments at different levels of plant organization raises an important question: how plastic is male gametophyte performance in response to given thermal conditions and to what extent does this plasticity allow plants to adjust their seed production to a changing environment? The range of opinions on this question varies from those who are convinced that the above-mentioned pollen–environment relationships are manifestations of genetically fixed responses (Mulcahy, 1979), to those who suggest that these differences are due to the plasticity of the gametophytic phenotype in response to prevailing temperatures during pollen development (Polito and Weinbaum, 1992; Havens, 1994). The current status of the pollen research community on this issue is that there is a plastic component of the pollen response to temperature that is at least partially affected by the environment during gametogenesis, but that the range of this plastic component of the phenotype may be limited within a given gametophyte (Stephenson and Bertin, 1983; Polito and Weinbaum, 1992; Pasonen et al., 2000). The scarcity of explicit tests on the sources of the variability in pollen performance to temperature precludes any generalizations except to say that more detailed studies are necessary.

## Pollen Studies: Past, Present, Future

### A Historical Overview of Pollen Studies

The putatively earliest records of pollen are contained in a prehistoric relief carving from the Palace of the Assyrian King Ashuir-nasir-pal II. (800 BC) and demonstrate early recognition of the sexes in plants (Knox, 1979). The very first scientific evidence for the importance of male gametophytes in plant sexual reproduction was possibly provided by Rudolf Camerarius, a German botanist working in the second half of the 17th century at the University of Tübingen. In his famous work on *Mercurialis*, *Ricinius*, and *Zea mais*, he demonstrated the important role of pollen as the main fertilizing agent. This pioneering work inspired others to carry out more observational and experimental studies on male gametophytes. Our understanding of pollination was later greatly advanced by Logan, Kölreuter, and Sprengel (Knox, 1979). At the beginning of the 19th century, the availability of new and more powerful microscopes with high magnification and high resolution allowed Turpin and Amici to discover the existence of the pollen tube. Mirbel, Hugo von Mohl, Fritsche, and Fischer made a great effort to develop methods for pollen morphology studies (particularly pollen wall structures) and developed the first classification of pollen grains based on their morphology. In the second half of the 19th century, the theory of evolution by natural selection caught on and naturalists (e.g., von Gärtner, Herbert, Hildebrand, and, of course, Darwin) carried out numerous experiments and observations on the operation of outcrossing mechanisms (Baker, 1983). At the end of the century, in 1897, double fertilization, the main target of the growing pollen tube into the style, was independently discovered by Nawaschin and Guignard (Jensen, 1998). During the early decades of the 20th century, botanists were quick to explore the effects of temperature and maternal nutrient supply on pollen performance, while evolutionary biologists began studying the nature of haploid selection and pollen competition (Williams and Mazer, 2016). Specifically, the experimental studies by Correns, Jones, Buchholz, and Blakeslee, and others indicated that pollen competition can strongly affect gene or chromosome frequencies (Williams and Mazer, 2016). In the 1940s, the rise of the synthetic study of evolution (also known as “neo-Darwinism”) gave pollen studies renewed importance. The works of Pijl, Faegri, Baker, Hurd, and Kevan described several pollination syndromes, due to their evolutionary importance for gene flow (Baker, 1983). In the middle of the last century, the continuous improvement of microscopy, especially the introduction of the electron microscope into pollen studies, in combination with the rapid development of highly precise analytical methods of physics and chemistry, advanced our knowledge of pollen ultrastructure, the pollen grain, and the pollen tube biochemistry and metabolism dramatically (Stanley and Linskens, 1974; Ottaviano et al., 1992; Heslop-Harrison, 2013). At the same time, there was a new surge of interest in the evolutionary and ecological aspects of male gametophyte development, particularly in the role of microgametophyte selection over pollen competition in plant adaptation and evolution (Mulcahy and Mulcahy, 1975; Mulcahy, 1979; Mulcahy and Ottaviano, 1983; Mulcahy et al., 1986; Ottaviano et al., 1992). These developments stimulated a new generation of research on pollen performance that has lasted through the beginning of the new millennium. Thanks to these scientists, most botanists now accept that both natural and sexual selection can affect post-pollination processes in the angiosperm (Williams and Mazer, 2016). It must also be noted that the whole field of pollen-related studies was revitalized in the late 1970s resulting in a very cohesive and productive research community that left behind a large body of knowledge on pollen morphology and its ultrastructure, pollen biology (including different aspects of microsporogenesis), pollen–pistil interactions and incompatibility (particularly evolution of plant breeding systems; Real, 1983; Dafni et al., 2000), and experimental methodologies with pollen (Stanley and Linskens, 1974; Knox, 1979; Mulcahy and Ottaviano, 1983; Mulcahy et al., 1986; Ottaviano et al., 1992; Mohapatra and Knox, 1996; Shivanna, 2003; Shivanna et al., 2005; Hesse et al., 2009; Clément et al., 2012; Heslop-Harrison, 2013).

The last two decades have been marked by vigorous research on the understanding of the molecular, biophysical, and physiological aspects of male gametophyte functioning at different stages of its development (anther – stigma – style – embryo sac). Briefly, serious progress has been achieved in describing the physiological and biochemical pathways of PG and PTG and identifying the genes encoding them (Palanivelu et al., 2003; Lalanne et al., 2004; Jiang et al., 2005; Dai et al., 2006). The recent advancement of imaging technology to monitor pollination and fertilization (e.g., intra-vital microscopy; Cheung et al., 2010; Ung et al., 2013; Vieira and Feijo, 2016) allows us to now better understand the molecular mechanisms of self-incompatibility (Staiger et al., 2010; Bedinger et al., 2017), signaling in PTG (Guan et al., 2013), kinetics and the economics of PTG (Hepler et al., 2013; Williams et al., 2016), as well as male–female cross-talk from PG on a stigma to double fertilization (Dresselhaus and Franklin-Tong, 2013; Higashiyama and Yang, 2017). At the higher levels of plant organization, further research on the “classic” topics of pollen biology studies, such as plant–pollinator interactions (the role of pollen limitation and heterospecific pollen transfer in seed siring success (Arceo-Gomez et al., 2016; Dafni and Vereecken, 2016; Wagner et al., 2016), plant mating systems (Cruzan and Barrett, 2016; Lankinen et al., 2016), and microgametophyte selection (Lankinen et al., 2013; Harder et al., 2016) has been done. The increasing awareness of the consequences of global climate change for the security of the world’s food supply has stimulated the interest of agriculturalists, particularly plant breeders to the responses of PG and PTG in cultivated plant species, such as rice, soybean, or cotton, to these new extreme temperature shifts (Kakani et al., 2005; Prasad P. et al., 2006; Salem et al., 2007; Mesihovic et al., 2016).

### Present Gaps

The contemporary landscape of pollen research includes a vast number of aspects of pollen biology and ecology. Yet, despite the concerted effort of the scientific community to discover a deeper and more refined understanding of male gametophyte functioning, there remain several gaps and biases in the existing understanding on the pollen–environment relationship in general, and the effects of temperature on pollen performance, in particular. These biases could be classified as follows.

#### Study Species Bias

The generally accepted extreme sensitivity of pollen to temperature stress rests mainly on the experiments carried out with domesticated plant species and their numerous cultivars (e.g., rice and cotton Matsui and Omasa, 2002; Kakani et al., 2005) or a very few indoor-cultivated model species, such as *Arabidopsis thaliana* (Boavida and McCormick, 2007). In contrast, studies on microgametophyte performance in wild species under different temperature conditions are extremely scarce and usually limited to very few number of taxa in studies where a large number of species were studied (Pigott and Huntley, 1981; McKee and Richards, 1998; Steinacher and Wagner, 2012; Sunmonu and Kudo, 2015), but see Rosbakh and Poschlod, 2016; Wagner et al., 2017. It is still not clear whether the results of such studies can be generalized into natural systems since a high number of mutants lacking of specific adaptations to the environmental conditions of the growing sites were used in the experimental populations (Stephenson and Bertin, 1983; Lyndon, 1992). In other words, it is not yet known how temperature affects pollen performance in wild plants, an important subject for future research.

The strong disproportion between studies done with cultivated and wild species is also reflected in the available methodology for pollen-related studies. The verified techniques for studying different aspects of the male gametophyte performance in wild species (with the exception of a couple of great handbooks on pollination (Kearns and Inouye, 1993; Dafni et al., 2005) are almost completely missing or limited to just a few species (Brewbaker and Kwack, 1963; Bassani et al., 1994; Speranza et al., 1997; Nepi et al., 2005), whereas there are numerous detailed and profound protocols for crop and model species (Shivanna, 2003; Shivanna et al., 2005; Boavida and McCormick, 2007).

#### “Stage” Bias

In cultivated and model plant species, all stages of male gametophyte development, from microsporogenesis to double fertilization, have received equal attention from the scientific community and consequently a comprehensive body of knowledge on each of them has been accumulated (see the references above). As for wild species, there is a clear deficit in understanding how the environment affects pollen performance on its way out of the anther toward the egg cell. Traditionally, pollen biology studies on wild species are mainly restricted to the mechanics of pollen dispersal (pollination; (Fenster et al., 2004; Faegri and Van der Pijl, 2013), plant mating systems (Barrett and Harder, 1996; Eckert et al., 2010), and pollen-pistil interactions (mechanism of self-compatibility; De Nettancourt, 2001), excluding any consideration of the interval between pollen deposition and fertilization (McKenna, 1986). The potential negative temperature effects on microsporogenesis, PG and PTG (Steinacher and Wagner, 2013; Rosbakh and Poschlod, 2016; Wagner et al., 2017), fertilization (Steinacher and Wagner, 2012), and, most importantly for the reproduction niche, their potential consequences for seed set (Wagner et al., 2012) are still poorly represented in the current landscape of pollen ecological research of natural populations. They definitely deserve more attention, as they too help to elucidate the details of pollen–environment relationships. The difficulty in studying these stages is perhaps due to their low accessibility (in fact, the male gametophyte is hidden in the anther or in the pistil, respectively), the above-mentioned lack of a method to study them should be remedied, and a larger sampling effort is required to apply a multispecies approach.

#### Aspect Bias

In last 20 years, several thousand studies on various aspects of pollen have been published. However, studies taking a molecular, physiological, or evolutionary approach to pollen functioning strongly outnumber those with a focus on the ecological and evolutionary aspects. There are, however, a few existing studies touching on the ecological aspects of temperature effects on male gametophyte functioning which clearly illustrate that knowledge on how altered pollen performance can help us to reach a better understanding of how the environment determines species characteristics, such as distributional limits and species abundancy because of its direct connection to the size and the quality of seed crops. As an early example, Pigott and Huntley (1981) demonstrated that the mismatch between the temperature requirements of PG and the ambient temperatures during the short period of stigmatic and stylar receptivity in *Tilia cordata* accounted for its northern distribution limit in the British Isles (see also Pigott and Warr, 1989; Pigott, 1992). As for the later example, Rosbakh and Poschlod (2016) suggested that the strong correlation between PG and PTG minimal temperature and habitat temperature, due to its direct effect on seeds, could in part explain recent changes in plant abundance in alpine vegetation (Gottfried et al., 2012; Pauli et al., 2012; Rosbakh et al., 2014). They speculate that the rise in temperature and the associated phenological shifts observed in the Alps in recent decades have lifted the restrictions of harsh high-altitude environments on sexual reproduction for lowland species. These species, which require relatively high temperatures to initiate PG and PTG, could benefit from the relaxation of the low-temperature filter by increasing seed production and contribute to range extension into alpine vegetation (Rosbakh and Poschlod, 2016).

To summarize, the ecological aspects of pollen performance in wild species, particularly the stages from microsporogenesis to gamete fusion are the “Achilles heel” of modern pollen-related studies and there is an urgent need for more studies on these issues.

## Conclusion and Perspectives

The regeneration niche (Grubb, 1977) has been an important concept in plant ecology for 40 years. Yet, the importance of the pre-fertilization stages for successful seed production has been largely ignored by the research community. In this review, we have demonstrated that these stages and its peculiarities, particularly the extreme sensitivity of male gametophyte performance to temperature, can be the key determinants of a species’ regeneration niche. Thus, we suggest that previous views stating that the regeneration niche begins with the production of seeds need to be modified to include the preceding stages. Yet, it seems that before we can achieve a better understanding on how the environment affects female and male gametophyte performance, we need to describe the ecophysiological mechanisms that determine this performance in various environments and evaluate the potential of altered performance on progeny growth and development. When it comes to the gaps in studies on the pollen-temperature relationship and its role on seed crops (see above), the main aim of this review, we propose that future studies go in the following directions. First, existing methodology on pollen ecophysiological studies should be generalized and become more available for plant biologists and ecologists dealing with uncultivated plant populations. There is still a need for a simple, yet effective, handbook of protocols which do not require sophisticated and expensive technology and could be applicable for a high number of plant taxa, allowing for fast screening of pollen traits for large sets of species. Easily implemented *in vitro* experiments on the effects temperature has on pollen (e.g., pollen longevity, PG, and PTG) could have a great potential to detect alterations in pollen performance at different developmental stages, as assays for defects in these parameters are difficult to perform *in vivo* (Stephenson and Bertin, 1983; Hedhly et al., 2005; Boavida and McCormick, 2007). Second, a deeper understanding of the variation of wild plant reproductive success under changing temperatures will require a body of work estimating thermal limits at different pollen developmental stages. Due to gene expression overlap between the gametophyte and sporophyte phases (e.g., Zamir et al., 1981), the results of such studies, apart their importance for estimating temperature tolerance of the reproduction niche, could be also used as a tool to estimate low- and high-temperature tolerance of adult plants (Hedhly, 2011). These data could be easily integrated into environmental and regeneration niche models as they can quantitatively estimate the success of reproduction under specific climatic conditions and are technically more easily measured than those relating to many other stages of the plant life cycle (Bykova et al., 2012; Rosbakh and Poschlod, 2016). Third, a tremendous amount of work remains to be done to accumulate data on the variation in pollen–temperature relationships at different levels of organization and identify the source (genetic or phenotypic) of this variation. Results of such studies could be used to estimate pollen performance plasticity and adaptability to changing temperature conditions, such as recent climate change. Finally, male gametophyte functioning in a changing environment has usually been studied from the perspective of a single component of its development. However, the temperature effects on pollen performance are more far-reaching and ostensibly affect the whole plant, thus determining its life-history (McKenna and Mulcahy, 1983; McKee and Richards, 1998). Therefore, the temperature effects on pollen performance should be followed through the entire life cycle, including seed germination, seedling establishment, as well as the performance of young and old individuals.

## Author Contributions

SR conceived the ideas and led the writing of the manuscript. All authors contributed critically to the drafts and gave the final approval for publication.

## Conflict of Interest Statement

The authors declare that the research was conducted in the absence of any commercial or financial relationships that could be construed as a potential conflict of interest.
